# The Relationship between Emotional Intelligence and Perception of Job Performance among Nurses in North West of Iran

**DOI:** 10.1155/2016/9547038

**Published:** 2016-06-28

**Authors:** Maryam Vahidi, Hossein Namdar Areshtanab, Mohammad Arshadi Bostanabad

**Affiliations:** Faculty of Nursing and Midwifery, Tabriz University of Medical Sciences, Tabriz 51745347, Iran

## Abstract

Emotional intelligence skills help nurses to cope with the emotional demands of healthcare environment. The aim of this study was to identify the relationship between emotional intelligence and perception of job performance among nurses. Using a correlational descriptive design with stratified random sampling, 338 registered nurses from teaching hospitals in North West of Iran were surveyed. Emotional intelligence and perception of job performance were measured using validated self-report measures. The collected data were analyzed through descriptive and inferential methods using SPSS/13. The mean of nurses' emotional intelligence and their perception of job performance was, respectively, 235.83 ± 37.98 and 157.63 ± 33.23. There was no significant relationship between nurses' emotional intelligence and their perception of job performance. Although there was a significant relationship between intrapersonal subscale of emotional intelligence and job performance, there was none with other subscales. In order to get rid of the physical and psychological effects of stressful work in wards, it seems that nurses just do routine activities and refuse working closely with the patients. It seems that fitting the patient to nurse ratio, dividing work between nurses, and supporting each other are necessary.

## 1. Introduction

Emotions are complex reaction patterns involving behavioral and physiological elements to personally significant events [1]. Almost in every experience there is an affectionate emotional aspect and managing it can have a significant role in general health and particular emotional health [2]. Paying attention to emotions, using them in human relationship, understanding oneself and others emotions, self-restraint, controlling instantaneous desires, sympathy with others, and using emotions in thinking, and understanding are among subjects discussed in the field of emotional intelligence [3].

Emotional labour has been widely accepted in the literature as a part of nursing work; while it is now considered acceptable for nurses to show their emotions as they empathize with patients and show their humanity, there is clearly a need for them to manage their emotions if they have to offer help and support [4]. Paying attention to the ability of nurses to accomplish their respective work goals, meet their expectations, achieve benchmarks, or attain their organizational goals is discussed as nursing performance [5]. Humanistic caring is nursing core [6]. Emotions are important in creating and maintaining a caring environment [7]. The nurse's ability to establish a rapport with patients, manage their own emotions, and empathize with patients is essential to provide quality care [4]. Most studies were done on relationship between emotional intelligence and academic performance among students which is different with job practice [7–11]. On the other hand, some studies have found no connection between these variables [12]. There are conflicting results about relationship between emotional intelligence and clinical performance. Also studies have shown that the job performance and emotional intelligence are affected by contextual factors [13]. The study aimed to examine the relationship between emotional intelligence and job performance among nurses.

## 2. Methods

### 2.1. Participants

The population of this correlational study was nurses in teaching hospitals of Tabriz University of Medical Sciences. The data for the study were collected during 2015. All nurses who met the inclusion criteria, absence of a known mental disease, at least one year of work experience, and informed consent to participate in the study, were recruited. A sample size of 338 participants was required to have 80% power and alpha 0.05 with using formula of sample size. The sampling was stratified random.

### 2.2. Instruments

In this study a three-part questionnaire was used. The first part of the questionnaire was related to personal-social characteristics of the nurses including age, sex, education level, marital status, and work experience. The second part was meant to measure emotional intelligence and the third part measured nurses' perception of job performance. To assess the emotional intelligence, we used Bar-On Questionnaire which includes 90 questions in 5 categories and 15 scales. The five categories are intrapersonal (self-regard, emotional self-awareness, assertiveness, independence, and self-actualization), interpersonal (empathy, social responsibility, and interpersonal relationship), stress management (stress tolerance and impulse control), adaptability (reality testing, flexibility, and problem solving), and general mood scale (optimism and happiness). Each question was designed based on a 5-point Likert scale scoring from 5 to 1 (completely agree = 5 to completely disagree = 1). The total score is the sum of all 14 scales scores. The minimum and maximum scores for each scale were 6 and 30, respectively, and the maximum and minimum total scores were 90 and 450, respectively. The questionnaire was standardized to be used in Iran; its content validity was reported to be acceptable and its reliability was 0.89 by test-retest method [14]. It was used many times in Tabriz and, therefore, the validity and reliability were not determined in this study. Scores were in the range of 90–450 points that total score lower than 270 was considered weak and more than 270 was good.

The six-dimension scale of nursing performance (6D Scale), a self-administered tool containing 52 four-point rating-scale items, evaluates nursing performance. All items are grouped into six performance subscales: leadership, critical care, teaching/collaboration, planning/evaluation, interpersonal relations and communications, and professional development. Each question was designed based on a 5-point Likert scale scoring from 5 to 1 (always = 5 to never = 1). The original reliability for this instrument yielded alpha values ranging from a high of 0.98 for the professional development scale to a low of 0.84 for the leadership scale in this study; back translation was used for validity of clinical competence questionnaire [7]. Its content validity was assessed by collaboration of ten faculty members in nursing and midwifery faculty. Its reliability was determined by test-retest and the correlation coefficient was determined 0.88 at two different times for participants. Scores were in the range of 52–260 points where total score lower than 156 was considered weak and more than 156 was good.

### 2.3. Statistical Analysis

The data were analyzed using SPSS 13. Descriptive statistics of the variables in study included frequency, percentage, means, and standard deviations. The association between the variables was initially explored using Pearson test, *t*-test, and ANOVA.

This study protocol was approved the Ethics Committee of Tabriz University of Medical Sciences (number 5.4.830). The study aims were explained and written informed consent was obtained from all nurses.

## 3. Results

In this study, 88.2 percent (298 nurses) of the total participants were female and 75.2 percent (254 nurses) were married. Mean age of nurses was 36.2 ± 7.1 years and mean of work experience was 11.75 ± 7.17 years. 92 percent (300 nurses) had B.S. degree, 4.9 percent (16 nurses) Associate degree, and 3.1 (10 nurses) M.S. degree. 69.6 percent (224 nurses) of them were working in circulation shift.

The results showed that the mean and standard deviation scores of nurses' emotional intelligence were 235.83 ± 37.98 which is less than average (mean = 270) and the mean and standard deviation scores of nurses' perception of job performance were 160.60 ± 29.21 which is greater than average (mean = 156). Relationship between emotional intelligence and nurses' perception of job performance was weak but it was not significant (*r* = 0.115, *p* = 0.139). The results of this study showed that there was no significant relationship between emotional intelligence, perception of job performance, and demographic characteristics of nurses (Table 1).

There was no significant correlation between emotional intelligence and nurses' perception of job performance with factors such as age, sex, marital status, education level, and working experience (Table 2).

## 4. Discussion

Finding of the study revealed that the mean score of nurses' emotional intelligence was weak. In this regard, in the study of Bakr and Safaan, Saeed et al., and Codier et al. the emotional intelligence score was reported to be of low level which was consistent with the results of the study [5, 15, 18]. However, several studies showed that nurses' emotional intelligence is moderate and high, which was not consistent with the results of our study [16–18]. Some differences may be due to cultural, educational differences in various communities and measurement by different instruments.

Based on the findings, the score of nurses' perception of job performance was above moderate. In this regard, in the study of AlMakhaita et al. and Kelley et al. the score of job performance was reported to be of high level which was consistent with the results of our study [19, 20]. However, several studies showed that nurses' perception of job performance is moderate and low, which was not consistent with the results of our study [5, 21]. Some differences may be due to cultural, organizational differences in various communities and measurement by different instruments.

Based on the findings, there was no significant relation between emotional intelligence and nurses' perception of job performance. In this regard, several studies showed that there was no significant relation between emotional intelligence and nursing performance which was consistent with the results of the study [22, 23]. However, several studies showed that there was significant relation between emotional intelligence and nurses' perception of job performance [12, 24–26]. This could be due to the nurses' work sitting that they have to work under system routines; so they do the same tasks every day. On the other hand, there is less chance to give their opinion in organizational decisions. Participation in organizational decision making is an important factor in nursing practice [27]. Finding of the study revealed that there was significant relation between intrapersonal dimension and nurses' perception of job performance. Intrapersonal dimension or self-awareness is important for nurses to know themselves well and it ultimately helps them to build a therapeutic environment of caring and healing [28].

Results of the study revealed that there was no significant relation between emotional intelligence and nurses' perception of job performance with factors as age, sex, marital status, education level, and the working experience. In the study of Vatankhah et al. and Hatamgooya et al., they reported that there was no significant relation between emotional intelligence and nurses' perception of job performance with the factors [29, 30]. These results remind us that emotional intelligence can be learned and developed at any marital status, education level, working experience age, and gender. However, several studies showed that there was significant relation between emotional intelligence and nurses' perception of job performance with the factors [31, 32]. It can be said that, with increasing age and work experience and ability to adapt with changes, personal and social problem solving also increase.

Some limitations of the study could be mentioned. The first is the size of the sample (i.e., 338). Use of self-report questionnaire may lead to an overestimation of some of the findings due to variance. Although finding a great amount of comparable teams within a single hospital is quite challenging, having larger sample size of teams would have strengthened the impact of the study's results.

## Figures and Tables

**Table 1:** Relationship between perception of job performance with nurses' emotional intelligence and its components.

*p* value	*r*	Mean ± SD	*N*	Variable
0.036^*∗*^	0.162	84.41 ± 15.43	338	Intrapersonal
0.738	−0.026	35.99 ± 8.84	338	Interpersonal
0.056	0.148	47.67 ± 8.52	338	Adaptability
0.187	0.102	36.19 ± 7.92	338	Stress management
0.959	0.004	31.55 ± 5.03	338	General mood
0.139	0.115	235.83 ± 37.98	338	Emotional intelligence
		160.60 ± 29.21	338	Perception of job performance

^*∗*^ *p* ≤ 0.05.

**Table 2:** Relationship between demographic factors with perception of job performance and nurses' emotional intelligence.

Job performance		Emotional intelligence		Demographic factors	
Statistical indicators	Mean (SD)	Statistical indicators	Mean (SD)		
*t* = −1.72	145.05 (45.27)	*t* = 0.57	240.52 (37.50)	Male	Gender
*p* = 0.86	157.46 (33.87)	*p* = 0.58	235.14 (38.37)	Female	

*t* = 0.01	157.71 (37.55)	*t* = −0.45	233.12 (37.83)	Single	Marital status
*p* = 0.99	157.70 (32.47)	*p* = 0.64	236.33 (38.25)	Married	

*F* = 0.88 *p* = 0.41	158.32 (51.98)	*F* = 0.98 *p* = 0.37	219.37 (63.52)	Associate degree	Educational status
	156.36 (32.65)		237.36 (36.32)	B.S degree	
	176.60 (33.63)		227.60 (36.78)	M.S. degree	

^*∗*^ *p* ≤ 0.05.
